# Salvage Transpapillary Biliary Drainage Using a Novel Device Delivery System via a PTBD‐Guided Rendezvous Technique

**DOI:** 10.1111/den.70054

**Published:** 2025-10-29

**Authors:** Tomoyuki Tanaka, Yasuhiro Kuraishi, Takaya Oguchi

**Affiliations:** ^1^ Department of Gastroenterology Shinshu University Hospital Matsumoto Japan; ^2^ Department of Gastroenterology Japan Red Cross Suwa Hospital Suwa Japan

## Abstract

Watch a video of this article.

The EndoSheather (Piolax, Kanagawa, Japan) is a novel device‐delivery system composed of a tapered inner catheter and a wide outer sheath [1]. It accommodates instruments up to 1.9 mm in diameter to enable stricture dilation and passage of large‐cup biopsy forceps, thereby facilitating mapping biopsies and troubleshooting procedures such as migrated stent retrieval [2, 3]. We herein describe the integration of the EndoSheather into a salvage percutaneous transhepatic biliary drainage‐guided rendezvous (PTBD‐RV) technique.

A 70‐year‐old man with a history of sigmoid colon neuroendocrine tumor resection suffered recurrent cholangitis secondary to malignant hilar bile duct stenosis from liver metastases (Figure 1). Tumor invasion had led to disconnection of the right anterior (Ba), right posterior (Bp) and left hepatic ducts. Plastic stents were placed in each segment, including one inserted into Segment 2 (B2).

**FIGURE 1:**
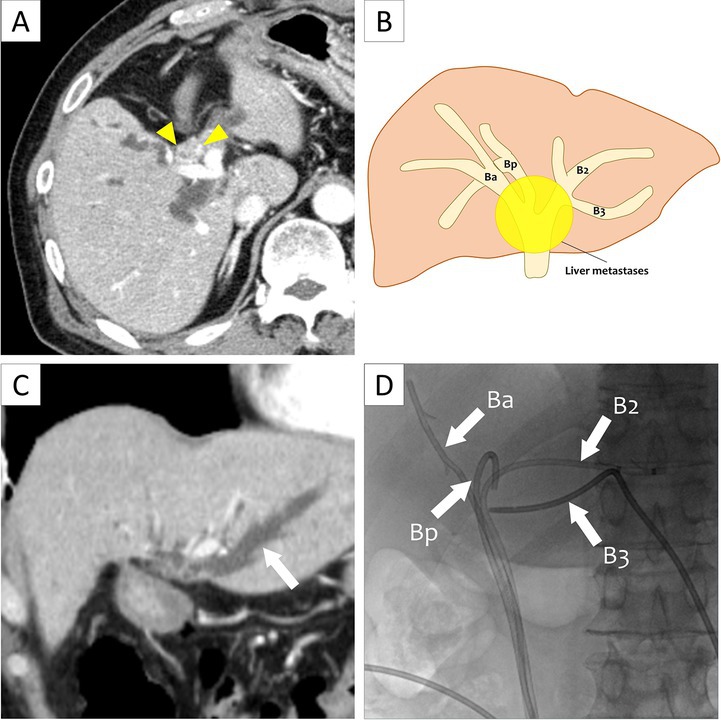
(A) The hilar bile duct exhibited stenosis due to metastatic lesions (yellow arrowheads). (B) Schematic illustration of biliary separation due to tumor invasion. Following tumor invasion from liver metastases, the right anterior duct (Ba), right posterior duct (Bp), and left hepatic duct were disconnected. Segment 2 (B2) and Segment 3 (B3) within the left hepatic duct were also nearly separated. (C) Computed tomography showed dilation of B3 (arrow). Tumor progression had caused disconnection between B2 and B3, which led to acute cholangitis in B3. (D) In addition to the previously placed stents in Ba, Bp, and B2, a percutaneous transhepatic biliary drainage (PTBD) tube was inserted into B3.

Two years later, cholangitis recurred in Segment 3 (B3). Following unsuccessful transpapillary guidewire access, a PTBD catheter was temporarily placed in B3 for urgent drainage due to severe cholangitis (Figure 1D). Transpapillary drainage was subsequently attempted using PTBD‐RV (Figure 2, Video S1).

**FIGURE 2:**
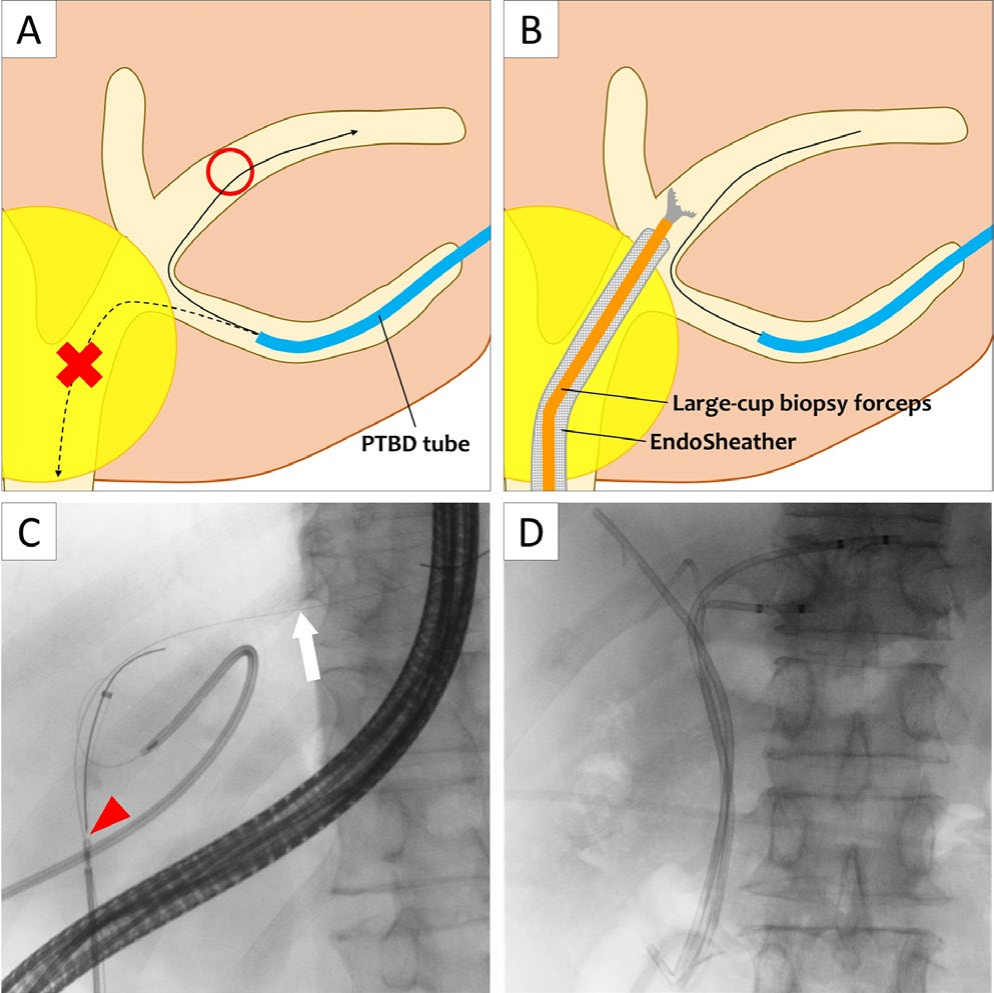
Schematic illustrations (A, B) and fluoroscopic images (C, D) of the PTBD‐guided rendezvous technique using the EndoSheather. (A) The guidewire inserted from the trans‐PTBD route could not pass through the stricture into the duodenum but was advanced into B2. (B) Another guidewire was inserted via the transpapillary route into B2, and the EndoSheather was advanced over this wire. Large‐cup biopsy forceps were delivered through the EndoSheather to grasp and retrieve the PTBD guidewire from B3, thereby establishing transpapillary access. (C) Before attempting to grasp the guidewire in B3, double guidewires were inserted into B2 through the EndoSheather to prevent accidental dislodgement. One of the guidewires was left in place (arrow), while the other was used to advance the EndoSheather. The large‐cup biopsy forceps were inserted through the EndoSheather, and the guidewire was successfully grasped by carefully adjusting both wire manipulation and the position of the opened forceps cups (red arrowhead). (D) Ultimately, plastic stents were successfully placed in B3, B2, Bp, and Ba, achieving complete drainage of all four ducts.

The guidewire from the PTBD route failed to traverse the stricture into the duodenum but reached B2. Another guidewire was introduced into B2 via the transpapillary route, and the EndoSheather was advanced over this wire across the stricture. Large‐cup biopsy forceps (Radial Jaw 4; Boston Scientific, MA, USA) were delivered through the sheath and grasped the PTBD guidewire in B2. The guidewire was withdrawn through the sheath to establish transpapillary access to B3. The tapered inner catheter enabled stricture traversal, while the wide‐lumen sheath facilitated forceps guidance. Plastic stents were successfully placed in B3, B2, Bp, and Ba, completing drainage of all four ducts.

PTBD‐RV is considered a salvage option for failed endoscopic retrograde cholangiopancreatography [4]. This case demonstrates the utility of the EndoSheather for complex malignant biliary strictures and transpapillary drainage [5].

## Author Contributions

Tomoyuki Tanaka wrote the manuscript. Yasuhiro Kuraishi co‐wrote and reviewed the manuscript. Takaya Oguchi supervised the endoscopy and reviewed the manuscript.

## Conflicts of Interest

The authors declare no conflicts of interest.

## Supporting information


**Video S1:** This case demonstrates the use of a novel device delivery system (EndoSheather) to facilitate percutaneous transhepatic biliary drainage‐guided rendezvous in a case of malignant hilar biliary obstruction. After conventional transpapillary guidewire passage failed due to severe bile duct stricture, the EndoSheather enabled the introduction of large‐cup biopsy forceps across the stenosis, allowing guidewire retrieval and successful transpapillary biliary stenting.
